# Prognostic Expression Signature of *RB1*, *PTEN*, and *TP53* Genes in Patients with Metastatic Hormone-sensitive Prostate Cancer Treated with Androgen Receptor Pathway Inhibitors

**DOI:** 10.1016/j.euros.2024.10.008

**Published:** 2024-10-21

**Authors:** Marta Garcia de Herreros, Natalia Jiménez, Leonardo Rodríguez-Carunchio, Eva Lillo, Mercedes Marín-Aguilera, Laura Ferrer-Mileo, Caterina Aversa, Samuel García-Esteve, Joan Padrosa, Isabel Trias, Laia Fernández-Mañas, Albert Font, Isabel Chirivella, Mariona Figols, Miguel Ángel Climent, Aleix Prat, Òscar Reig, Begoña Mellado

**Affiliations:** a Translational Genomics and Targeted Therapeutics in Solid Tumours Lab, Fundació de Recerca Clínic Barcelona-Institut d’Investigacions Biomèdiques August Pi i Sunyer, Barcelona, Spain; b Medical Oncology Department, Hospital Clínic, Barcelona, Spain; c Uro-Oncology Unit, Hospital Clínic, University of Barcelona, Barcelona, Spain; d Department of Pathology, Hospital Clínic, Barcelona, Spain; e Department of Medicine, University of Barcelona, Barcelona, Spain; f Centro de Investigación Biomédica en Red de Enfermedades Raras, Instituto de Salud Carlos III, Madrid, Spain; g Medical Oncology Department, Institut Català d’Oncologia, Hospital Germans Trias i Pujol, Badalona, Spain; h Department of Medical Oncology, Hospital Clínico Universitario de Valencia, Instituto de Investigación Sanitaria (INCLIVA), University of Valencia, Valencia, Spain; i Medical Oncology Department, Fundació Althaia, Xarxa Assistencial Universitària de Manresa, Manresa, Spain; j Medical Oncology Service, Instituto Valenciano de Oncología (IVO), Valencia, Spain

**Keywords:** Hormone-sensitive prostate cancer, Tumor suppressor genes, Biomarkers, Androgen deprivation therapy, Androgen receptor pathway inhibitors, Aggressive-variant prostate cancer

## Abstract

Alterations in the tumor suppressor genes (TSGs) *RB1*, *PTEN*, and *TP53* are associated with treatment resistance, worse survival, and aggressive variants of prostate cancer (AVPC). We previously developed and validated a signature reflecting low TSG expression (TSG_low_) that was associated with poor outcomes in patients with metastatic hormone-sensitive prostate cancer (mHSPC) treated with androgen deprivation therapy (ADT) ± docetaxel. The aim of this multicenter retrospective study was to validate the TSG_low_ signature in patients with mHSPC treated with ADT and an androgen receptor pathway inhibitor (ARPI) and to explore clinical characteristics at progression according to TSG status. TSG mRNA expression in formalin-fixed, paraffin-embedded samples was assessed via nCounter. Correlation of expression levels with castration-resistant prostate cancer–free survival (CRPC-FS; primary endpoint) and overall survival (OS) was investigated via Kaplan-Meier and multivariate Cox analyses. Of the 137 patients included, 77.4% had de novo stage IV cancer and 44.5% had high-risk disease. TSG_low_ (16.8%) was correlated with visceral metastases (*p* = 0.013), high-risk disease (*p* = 0.038), higher Gleason score (*p* = 0.026), shorter CRPC-FS (hazard ratio 1.9; *p* = 0.046) and higher AVPC frequency (*p* = 0.01). Our results confirm that a TSG_low_ signature is an adverse prognostic factor and is associated with AVPC development in patients with mHSPC treated with ADT + ARPI. Further prospective validation is needed to define specific therapeutic strategies for these patients.

**Patient summary:**

We looked at outcomes for patients with metastatic hormone-sensitive prostate cancer treated with hormone therapies. We found that patients with low expression of two out of three tumor suppressor genes (*TP53*, *RB1*, *PTEN*) had worse clinical outcomes and had aggressive variants of prostate cancer. Measuring the expression of these genes in early-stage prostate cancer could help in finding better treatments for these patients.

Prostate cancer (PC) is ranked second in incidence and fifth in mortality among cancers in men. Androgen deprivation therapy (ADT) with a novel androgen receptor pathway inhibitor (ARPI), and ADT + ARPI + docetaxel are standard upfront treatments in metastatic hormone-sensitive prostate cancer (mHSPC) [1], [2].

Alterations in the tumor suppressor genes (TSGs) *RB1, PTEN,* and *TP53* are associated to aggressive variants of prostate cancer (AVPC) and neuroendocrine (NE) dedifferentiation in castration-resistant prostate cancer (CRPC), as well as poor prognosis and reduced response to conventional treatments [3], [4]. In mHSPC, about 30% of patients exhibit mutations in at least one TSG and are associated with poor clinical outcomes [5]. Besides, the transcriptional profile of primary tumors may determine a distinct clinical evolution of mHSPC patients. A transcriptional analysis of the STAMPEDE clinical trial in patients treated with ADT with or without abiraterone, identified that PTEN and TP53 loss signatures correlated with adverse prognosis [6].

Our prior work revealed that low TSG expression correlated with exome mutations and low immunohistochemistry expression and had better accuracy than other models in predicting adverse clinical outcomes [7]. We developed and validated a TSG_low_ signature in patients with mHSPC treated with ADT or ADT + docetaxel that was associated with lower AR expression and shorter CRPC-free survival (CRPC-FS) and overall survival (OS) [7]. In the current study, we validated the TSG_low_ signature in a series of patients with mHSPC who received ADT + ARPI as standard therapy.

Our multicenter retrospective biomarker study included patients with mHSPC treated with ADT (luteinizing hormone–releasing hormone analog) + ARPI (abiraterone 1000 mg/d + prednisone 5 mg/d, or enzalutamide 160 mg/d, or apalutamide 240 mg/d). Key inclusion criteria were a diagnosis of prostate adenocarcinoma and the availability of formalin-fixed, paraffin-embedded (FFPE) biopsy tissue from either the primary tumor or metastases in the hormone-sensitive setting for molecular analysis.

Patients with NE prostate cancers were excluded. Clinical data were collected from electronic records. Clinical features of AVPC were collected at CRPC progression. AVPC was defined as the presence of at least one of the following: exclusively visceral metastases, predominantly lytic metastases, bulky nodal or local disease, low prostate-specific antigen, NE markers, or a short interval to CRPC following ADT initiation [4]. TSG expression in FFPE tumor samples was assessed via nCounter as previously described [7]. Low TSG expression (TSG_low_) was defined as expression below the pre-established cutoff for at least two of the three TSGs [7]. The remaining cases were classified as wild-type TSG (TSG_wt_). The Kaplan-Meier method and the log-rank test were used to evaluate correlation of the TSG signature with CRPC-FS (primary endpoint) from the date of ADT initiation to the time of CRPC development, and with OS from the date of ADT initiation to the time of death or last follow-up visit. Univariate and multivariate Cox regression analyses were performed. The methods are described in detail in the Supplementary material.

A total of 137 patients were included, of whom 99 were treated with abiraterone, 35 with enzalutamide, and three with apalutamide. Baseline characteristics are summarized in Table 1. The group of 23 patients classified as TSG_low_ (16.8%) had higher frequencies of visceral metastases (*p* = 0.013), high-risk disease (*p* = 0.038), and higher Gleason scores (*p* = 0.026). Median follow-up was 30.7 mo (range 2.4–91.3), during which 48 patients (35%) developed CRPC and 56 (40.9%) died.

**Table 1:** Patient characteristics in the overall cohort and by TSG expression group

	Overall cohort	TSG_low_	TSG_wt_	*p* value a
Patients, *n* (%)	137	23 (16.8)	114 (83.2)	
Median age, yr (range)	71.8 (50.1–92.8)	71.1 (54.9–82.6)	72.4 (50.1–92.8)	0.888
Median PSA at diagnosis, ng/ml (range)	30 (1.8–4600)	16 (3.1–4600)	33 (1.8–3226)	0.542
ECOG PS score, *n* (%)				
0	42 (30.7)	6 (26.1)	36 (31.6)	0.064
1–2	82 (59.9)	17 (73.9)	65 (57)	
Data not available	13 (9.5)	–	13 (11.4)	
Stage at diagnosis, *n* (%)				
Stage <IV	28 (20.4)	3 (13)	25 (21.9)	0.406
Stage IV	106 (77.4)	20 (87)	86 (75.4)	
Data not available	3 (2.2)	–	3 (2.6)	
Gleason sum score at diagnosis, *n* (%)				
≤7	31 (22.6)	1 (4.3)	30 (26.3)	**0.026**
≥8	103 (75.2)	21 (91.3)	82 (71.9)	
Data not available	3 (2.2)	1 (4.3)	2 (1.8)	
Presence of visceral metastases, *n* (%)				
Yes	22 (16.1)	8 (34.8)	14 (12.1)	**0.013**
No	115 (83.9)	15 (65.2)	100 (87.7)	
Disease volume, *n* (%)				
High	73 (53.3)	16 (69.6)	57 (50)	0.11
Low	64 (46.7)	7 (30.4)	57 (50)	
Risk, *n* (%)				
High	61 (44.5)	15 (65.2)	46 (40.4)	**0.038**
Low	76 (55.5)	8 (34.8)	68 (59.6)	

ECOG PS = Eastern Cooperative Oncology Group performance status; PSA = prostate-specific antigen; TSG = tumor suppressor gene (*RB1*, *PTEN*, *TP53*); TSG_low_ = low expression of at least two of *RB1*, *PTEN*, and *TP53*; wt = wild-type.

a *p* values are for Fisher’s exact test for categorical variables or a Wilcoxon Mann–Whitney *U* test for continuous variables. Significant *p* values (<0.05) are indicated in bold font.

TSG_low_ was correlated with shorter CRPC-FS (hazard ratio [HR] 1.9, 95% confidence interval [CI] 1–3.6; *p* = 0.046; Fig. 1A,B). There was no significant difference in OS between the TSG groups (Fig. 1C,D). Further exploration of pairwise TSG combinations revealed shorter CRPC-FS for patients with low *RB1* and *PTEN* expression (HR 2.7, 95% CI 1.2–6.1; *p* = 0.011) and for patients with low *RB1* and *TP53* expression (HR 4.8, 95% CI 2.2–10.8; *p* < 0.001). Only concomitant low *RB1* and *PTEN* expression was correlated with shorter OS (HR 2.5, 95% CI 1.2–5.3; *p* = 0.017; Supplementary Fig. 1). Moreover, RB1_low_-PTEN_low_ (HR 5.1, 95% CI 1.9–14; *p* = 0.001) and RB1_low_-TP53_low_ (HR 3.9, 95% CI 1.4–10.6; *p* = 0.008) were independently correlated with shorter CRPC-FS, and RB1_low_-PTEN_low_ was independently correlated with shorter OS (HR 3.2, 95% CI 1.2–8.5; *p* = 0.021; Supplementary Fig. 2).

**Fig. 1:**
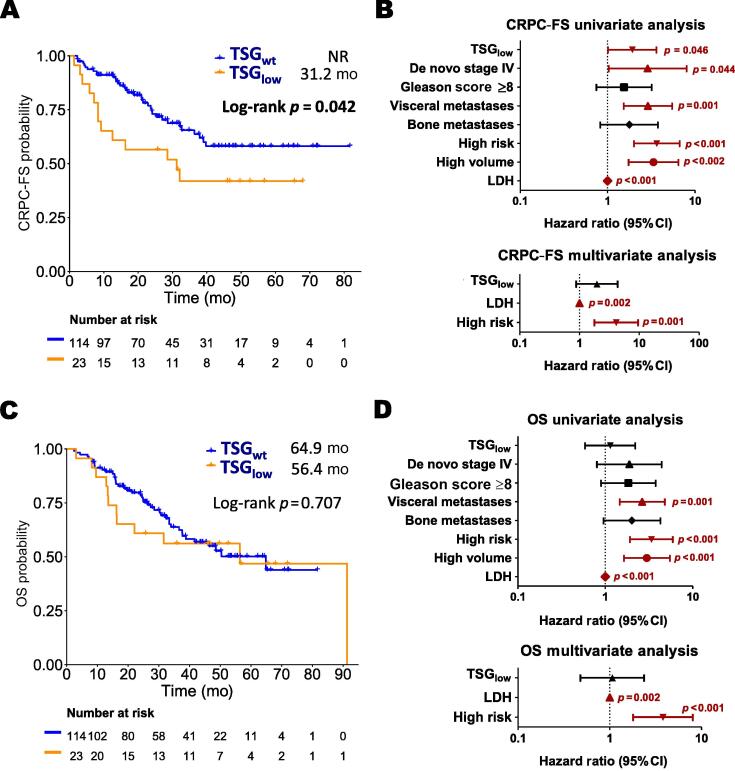
(A) Kaplan-Meier CRPC-FS curve by TSG expression, with median results shown. (B) Forest plots of univariate and multivariate analysis results for CRPC-FS. (C) Kaplan-Meier OS curve by TSG expression, with median results shown. (D) Forest plots of univariate and multivariate analysis results for OS. Significant *p* values (<0.05) are indicated in bold font. CI = confidence interval; CRPC-FS = castration-resistant prostate cancer–free survival; LDH = lactate dehydrogenase; NR = not reached; OS = overall survival; TSG = tumor suppressor gene.

Among the 48 patients who developed CRPC, clinical data at the time of CRPC progression were available for 42 (87.5%), and 13 (27.1%) met at least one AVPC criterion. The incidence of AVPC was significantly higher for the CRPC subgroup with TSG_low_ tumors (*n* = 9, 21.9%) than for the CRPC subgroup with TSG_wt_ tumors (66.7% vs 18.7%; *p* = 0.01; Supplementary Table 1). Five patients (10.4%) were classified as RB1_low_-PTEN_low_ and all of them had AVPC features at CRPC (Supplementary Tables 1–4). A biopsy was performed at the time of CRPC in four patients, with NE differentiation observed in all of the biopsies (Supplementary Fig. 3).

In conclusion, low TSG expression was correlated with early CRPC progression in a cohort of patients treated with ADT + ARPI, and was associated with the development of AVPC. These results confirm the adverse prognostic value of the TSG_low_ signature in patients with mHSPC [7] receiving different frontline treatments (ADT alone, ADT + docetaxel, or ADT + ARPI). Notably, only the RB1_low_-PTEN_low_ combination independently correlated with shorter CRPC-FS and OS, and all the patients with RB1_low_-PTEN_low_ status developed AVPC. The RB1_low_-TP53_low_ combination was also independently correlated with CRPC-FS. Alterations in these three genes are commonly found in NE prostate cancer, which may explain the adverse clinical evolution of their combinations [8]. Whether different TSG alterations are associated with different molecular and clinical outcomes will require further investigation. Data on family history and other demographic characteristics such as ethnicity would also enhance the characterization of patients with this profile.

We showed that patients with mHSPC which had TSG_low_ tumors have aggressive features, such as a greater frequency of visceral metastasis, higher Gleason scores, and high-risk disease. Besides, at CRPC progression, the group of patients with TSG_low_ tumors had a higher frequency of AVPC. Thus, assessment of this TSG signature in noncastrated tumors may represent a useful molecular biomarker for predicting tumor aggressiveness at CRPC and adverse outcomes. Patients harboring AVPC are a recognizable subset characterized by aggressive behavior, poor prognosis, and good response to platinum-containing chemotherapies [4]. Identification of patients who are likely to develop these AVPC earlier in the hormone-sensitive stage may be useful for predicting clinical outcomes, scheduling closer monitoring, and tailoring better treatment strategies. Early treatment with platinum [4], [9] and targeted therapies such as AKT inhibitors [10] are options that should be tested in clinical trials for these patients.

In conclusion, the TSG_low_ signature is an adverse prognostic factor for CRPC-FS and was associated with AVPC development in patients with mHSPC treated with ADT + ARPI. Further prospective validation is needed to define distinct therapeutic strategies for these patients.

  ***Author contributions*:** Begoña Mellado had full access to all the data in the study and takes responsibility for the integrity of the data and the accuracy of the data analysis.

  *Study concept and design*: Garcia de Herreros, Jiménez, Reig, Mellado.

*Acquisition of data*: Garcia de Herreros, Jiménez, Rodríguez-Carunchio, Lillo, Marín-Aguilera, Ferrer-Mileo, Aversa, Trias, Fernández-Mañas, Font, Chirivella, Figols, Climent, Reig, Mellado.

*Analysis and interpretation of data*: Garcia de Herreros, Jiménez, Rodríguez-Carunchio, Ferrer-Mileo, Aversa, García-Esteve, Padrosa, Reig, Mellado.

*Drafting of the manuscript*: Garcia de Herreros, Jiménez, Reig, Mellado.

*Critical revision of the manuscript for important intellectual content*: All authors.

*Statistical analysis*: Garcia de Herreros, Jiménez, Padrosa, Reig, Mellado.

*Obtaining funding*: Mellado.

*Administrative, technical, or material support*: Garcia de Herreros, Jiménez, Rodríguez-Carunchio, Marín-Aguilera, Ferrer-Mileo, Aversa, García-Esteve, Trias, Fernández-Mañas, Prat, Reig, Mellado.

*Supervision*: Mellado.

*Other*: None.

  ***Financial disclosures:*** Begoña Mellado certifies that all conflicts of interest, including specific financial interests and relationships and affiliations relevant to the subject matter or materials discussed in the manuscript (eg, employment/affiliation, grants or funding, consultancies, honoraria, stock ownership or options, expert testimony, royalties, or patents filed, received, or pending), are the following: Marta Garcia de Herreros reports speaker honoraria from Ipsen and travel and accommodation expenses from Novartis. Laura Ferrer-Mileo reports speaker honoraria and travel and accommodation expenses from Pfizer and Kyowa Kirin, and research funding from Roche. Caterina Aversa reports speaker honoraria from BMS, Janssen, and Pfizer, and travel expenses from Janssen. Albert Font reports research funding from AstraZeneca; a consulting or advisory role for Janssen, Astellas, and Bayer; and travel and accommodation expenses from Janssen. Isabel Chirivella reports advisory board participation for Pfizer, EISAI, and BMS. Mariona Figols reports speaker bureau particpation for Pfizer, Ipsen, and Astellas, and travel and accommodation expenses from Merck. Miguel Ángel Climent reports a consulting or advisory role for BMS, MSD, Bayer, EUNSA, Pfizer, Roche, Janssen, Pierre Fabre, and Ipsen; and travel and accommodation expenses from Janssen, Astellas, Roche, Ipsen, and MSD. Aleix Prat reports advisory and consulting fees from Roche, Pfizer, Novartis, Amgen, BMS, Puma, Oncolytics Biotech, MSD, Guardant Health, Peptomyc, and Lilly; lecture fees from Roche, Pfizer, Novartis, Amgen, BMS, Daiichi Sankyo, and Nanostring Technologies; institutional financial interests in Boehringer, Novartis, Roche, Nanostring Technologies, Sysmex Europe GmbH, Medica Scientia Innovation Research, SL, Celgene, Astellas, and Pfizer; a leadership role in Reveal Genomics and SL; and a patent interest in PCT/EP2016/080056. Òscar Reig reports a consulting or advisory role for BMS, EISAI, and Ipsen, and travel and accommodation expenses from Ipsen and Pfizer. Begoña Mellado reports research funding from Janssen, Roche, Bayer, and Pfizer; speaker bureau participation for Roche, Sanofi, Janssen, Astellas, Pfizer, Novartis, and Bristol-Myers Squibb; and travel and accommodation expenses from Janssen and Pfizer. The remaining authors have nothing to disclose.

  ***Funding/Support and role of the sponsor*:** This work was supported by Instituto de Salud Carlos III-Subdirección General de Evaluación y Fomento de la Investigación (PI18/714) and was co-funded by the European Union. Institutional funding from CERCA Programme/Generalitat de Catalunya is gratefully acknowledged. Marta Garcia de Herreros is supported by Contracte Clínic Recerca Emili Letang i Josep Font 2023. Òscar Reig is the recipient of an Ayudas SEOM de Intensificación para Investigadores Jóvenes award from the Spanish Society of Medical Oncology (SEOM). This work was developed at CELLEX, Barcelona, Spain. The sponsors played no direct role in the study.

  ***Data sharing statement*:** The Nanostring nCounter gene expression data presented in this study are available on reasonable request from the corresponding author and authorization from the institution.

  ***Ethics considerations*:** This study was conducted in accordance with the principles of the Declaration of Helsinki and was approved by institutional ethics committees of all participating centers. All patients provided signed informed consent before their inclusion in the study.

  ***Acknowledgments*:** We would like to thank the IGTP-HUGTP Biobank (PT13/0010/0009, PT17/0015/0045), a member of the Spanish National Biobanks Network and the Tumour Bank Network of Catalonia, and BioBank FIVO (PT17/0015/0051), a member of the Spanish National Biobanks Network and the Valencian Biobanking Network, for their collaboration in providing samples. We are also deeply indebted to all the patients who agreed to participate in the study.

## References

[1] Fizazi K., Foulon S., Carles J. (2022). Abiraterone plus prednisone added to androgen deprivation therapy and docetaxel in de novo metastatic castration-sensitive prostate cancer (PEACE-1): a multicentre, open-label, randomised, phase 3 study with a 2 × 2 factorial design. Lancet.

[2] Smith M.R., Hussain M., Saad F. (2022). Darolutamide and survival in metastatic, hormone-sensitive prostate cancer. N Engl J Med.

[3] Aparicio A.M., Shen L., Tapia E.L.N. (2016). Combined tumor suppressor defects characterize clinically defined aggressive variant prostate cancers. Clin Cancer Res.

[4] Aparicio A.M., Harzstark A.L., Corn P.G. (2013). Platinum-based chemotherapy for variant castrate-resistant prostate cancer. Clin Cancer Res.

[5] Velez M.G., Kosiorek H.E., Egan J.B. (2022). Differential impact of tumor suppressor gene (TP53, PTEN, RB1) alterations and treatment outcomes in metastatic, hormone-sensitive prostate cancer. Prostate Cancer Prostat Dis.

[6] Attard G, Parry M, Grist E, et al. Clinical testing of transcriptome-wide expression profiles in high-risk localized and metastatic prostate cancer starting androgen deprivation therapy: an ancillary study of the STAMPEDE abiraterone phase 3 trial. Research Square preprint. 10.21203/rs.3.rs-2488586/v1.

[7] Jiménez N., Garcia De Herreros M., Reig Ò. (2024). Development and independent validation of a prognostic gene expression signature based on RB1, PTEN, and TP53 in metastatic hormone-sensitive prostate cancer patients. Eur Urol Oncol.

[8] Beltran H., Prandi D., Mosquera J.M. (2016). Divergent clonal evolution of castration-resistant neuroendocrine prostate cancer. Nat Med.

[9] Stefàno E., De Castro F., Ciccarese A. (2024). An overview of altered pathways associated with sensitivity to platinum-based chemotherapy in neuroendocrine tumors: strengths and prospects. Int J Mol Sci.

[10] Sweeney C., Bracarda S., Sternberg C.N. (2021). Ipatasertib plus abiraterone and prednisolone in metastatic castration-resistant prostate cancer (IPATential150): a multicentre, randomised, double-blind, phase 3 trial. Lancet.

