# Supplementary material for: Prognostic Expression Signature of RB1, PTEN, and TP53 Genes in Patients with Metastatic Hormone-sensitive Prostate Cancer Treated with Androgen Receptor Pathway Inhibitors
Source: Eur Urol Open Sci. 2024 Oct 21;70:86–90. doi: 10.1016/j.euros.2024.10.008 (PMC11536031; doi:10.1016/j.euros.2024.10.008)
Supplement: Supplementary Data 1 [file mmc1.docx]

**Supplementary material**

**Supplementary methods**

***Design of the gene expression panel***

We configured an nCounter expression panel (Nanostring Technologies, Seattle, WA, USA) for 184 genes comprising signatures related to the development of castration-resistant prostate cancer (CRPC) and androgen suppression or taxane resistance [1]. Here, we present data for the TSG (*RB1*, *PTEN*, and *TP53*) signature.

***RNA extraction***

Sections of formalin-fixed, paraffin-embedded (FFPE) tumor tissue prepared as previously described [1] were stained with hematoxylin and eosin to determine the tumor area. Total RNA was extracted from at least two 10-μm macrodissected FFPE slides using an AllPrep DNA/RNA FFPE Kit (Qiagen, Hilden, Germany) according to the manufacturer’s protocol. RNA was quantified on a Nanodrop ND-1000 spectrophotometer (Thermo Scientific, Wilmington, MA, USA).

***nCounter analysis of gene expression***

A minimum of ~100 ng of total RNA was used to measure gene expression on an nCounter platform according to the manufacturer’s protocol (Nanostring Technologies, Seattle, WA, USA). RNA was hybridized into 192 probe sets for 18 h at 67°C and processed as previously described [1]. Raw expression counts were collected, normalized, and log_2_-transformed using nSolver 4.0 (RRID:SCR_003420) [1].

***Bioinformatics and statistical analysis***

A cutoff previously established and validated by our group [2] was applied to transformed z-scores for nCounter gene expression data for *RB1*, *PTEN*, and *TP53*. Cases were categorized as low or wild-type (wt) according expression levels in relation the cutoff for each gene.

CRPC-FS definition, treatment-response criteria and progressive disease were defined following the Prostate Cancer Clinical Trials Working Group 2 criteria [3].

Clinical parameters such as de novo stage IV disease, Gleason at diagnosis, the presence of visceral metastasis, bone metastasis, risk (as defined in LATITUDE [4]), and volume (as defined in CHAARTED [5]) at initiation of androgen deprivation therapy (ADT) were evaluated as dichotomous variables. Lactate dehydrogenase at ADT initiation was evaluated as a continuous variable.

Fisher’s exact test and the Wilcoxon Mann-Whitney *U* test were used for between-group comparison of proportions for categorical and continuous variables, respectively. Analyses were performed with R version 4.1.2 (R Foundation for Statistical Computing, Vienna, Austria).

**References**

[1] Jiménez N, Reig Ò, Marín-Aguilera M, et al. Transcriptional profile associated with clinical outcomes in metastatic hormone-sensitive prostate cancer treated with androgen deprivation and docetaxel. Cancers 2022;14:4757. https://doi.org/10.3390/cancers14194757

[2] Jiménez N, Garcia De Herreros M, Reig Ò, et al. Development and independent validation of a prognostic gene expression signature based on RB1, PTEN, and TP53 in metastatic hormone-sensitive prostate cancer patients. Eur Urol Oncol 2024;7:954–64. https://doi.org/10.1016/j.euo.2023.12.012

[3] Scher HI, Halabi S, Tannock I, et al. Design and end points of clinical trials for patients with progressive prostate cancer and castrate levels of testosterone: recommendations of the Prostate Cancer Clinical Trials Working Group. J Clin Oncol 2008;26:1148–59. https://doi.org/10.1200/JCO.2007.12.4487

[4] Fizazi K, Tran N, Fein L, et al. Abiraterone plus prednisone in metastatic, castration-sensitive prostate cancer. N Engl J Med 2017;377:352–60. https://doi.org/10.1056/NEJMoa1704174.

[5] Sweeney CJ, Chen YH, Carducci M, et al. Chemohormonal therapy in metastatic hormone-sensitive prostate cancer. N Engl J Med 2015;373:737–46. https://doi.org/10.1056/NEJMoa1503747

**Supplementary tables**

**Supplementary Table 1 – AVPC status at castration-resistant prostate cancer by TSG expression**

| Parameter | Patients, *n* (%) | | | *p* value ^a^ |
| --- | --- | --- | --- | --- |
|  | Overall cohort | TSG_low_ | TSG_wt_ |  |
| Patients | 42 | 9 (21.9) | 32 (78.1) |  |
| Meets the AVPC criteria   Yes   No | 13 (30.9)  29 (69.1) | 6 (66.7)  3 (33.3) | 6 (18.7)  26 (81.3) | **0.01** |
| Visceral metastasis only | 3 (7.1) | 2 (22.2) | 1 (3.1) | 0.11 |
| Lytic bone metastasis | 5 (11.9) | 2 (22.2) | 3 (9.4) | 0.28 |
| Bulky nodes or prostate mass | 7 (16.7) | 4 (44.4) | 3 (9.4) | **0.024** |
| Low PSA relative to volume | 4 (9.5) | 2 (22.2) | 2 (6.2) | 0.13 |
| NE markers + elevated LDH or CEA | 5 (11.9) | 1 (11.1) | 4 (12.5) | 0.43 |
| Primary castration resistance | 3 (7.1) | 1 (11.1) | 2 (6.2) | 0.53 |
| Small cell carcinoma morphology | 4 (9.5) | 1 (11.1) | 3 (9.4) | 0.7 |

AVPC = aggressive-variant prostate cancer; CEA = carcinoembryonic antigen; LDH = lactate dehydrogenase; NE = neuroendocrine; PSA = prostate-specific antigen; TSG = tumor suppressor gene; wt = wild-type.

^a^ *p* values are for Fisher’s exact test. Significant values (*p* < 0.05) are indicated in bold font.

**Supplementary Table 2 – AVPC status at castration-resistant prostate cancer by *RB1* and *PTEN* expression**

| Parameter | Patients, *n* (%) | | | *p* value ^a^ |
| --- | --- | --- | --- | --- |
|  | Overall cohort | RB1_low_-PTEN_low_ | Other |  |
| Patients | 42 | 5 (12.1) | 36 (87.9) |  |
| Meets the AVPC criteria   Yes   No | 13 (30.9)  29 (69.1) | 5 (100)  0 (0) | 7 (19.5)  29 (80.5) | **0.001** |
| Visceral metastasis only | 3 (7.1) | 1 (20) | 2 (5.6) | 0.33 |
| Lytic bone metastasis | 5 (11.9) | 1 (20) | 4 (11.1) | 0.45 |
| Bulky nodes or prostate mass | 7 (16.7) | 2 (40) | 5 (13.9) | 0.12 |
| Low PSA relative to volume | 4 (9.5) | 1 (20) | 3 (8.4) | 0.23 |
| NE markers + elevated LDH or CEA | 5 (11.9) | 1 (20) | 4 (11.1) | 0.45 |
| Primary castration resistance | 3 (7.1) | 1 (20) | 2 (5.6) | 0.33 |
| Small-cell carcinoma morphology | 4 (9.5) | 1 (20) | 3 (8.4) | 0.23 |

AVPC = aggressive-variant prostate cancer; CEA = carcinoembryonic antigen; LDH = lactate dehydrogenase; NE = neuroendocrine; PSA = prostate-specific antigen.

^a^ *p* values are for Fisher’s exact test. Significant values (*p* < 0.05) are indicated in bold font.

**Supplementary Table 3 – AVPC status at castration-resistant prostate cancer by *RB1* and *TP53* expression**

| Parameter | Patients, *n* (%) | | | *p* value ^a^ |
| --- | --- | --- | --- | --- |
|  | Overall cohort | RB1_low_-TP53_low_ | Other |  |
| Patients | 42 | 4 (12.1) | 37 (87.9) |  |
| Meets the AVPC criteria   Yes   No | 13 (30.9)  29 (69.1) | 3 (75)  1 (25) | 9 (24.3)  28 (75.7) | 0.56 |
| Visceral metastasis only | 3 (7.1) | 1 (25) | 2 (5.4) | 0.27 |
| Lytic bone metastasis | 5 (11.9) | 1 (25) | 4 (10.8) | 0.35 |
| Bulky nodes or prostate mass | 7 (16.7) | 2 (50) | 5 (13.5) | 0.081 |
| Low PSA relative to volume | 4 (9.5) | 1 (25) | 3 (8.1) | 0.13 |
| NE markers + elevated LDH or CEA | 5 (11.9) | 1 (25) | 4 (10.8) | 0.35 |
| Primary castration resistance | 3 (7.1) | 1 (20) | 2 (5.6) | 0.33 |
| Small-cell carcinoma morphology | 4 (9.5) | 1 (25) | 3 (8.1) | 0.29 |

AVPC = aggressive-variant prostate cancer; CEA = carcinoembryonic antigen; LDH = lactate dehydrogenase; NE = neuroendocrine; PSA = prostate-specific antigen.

^a^ *p* values are for Fisher’s exact test.

**Supplementary Table 4 – AVPC status at castration-resistant prostate cancer by PTEN and TP53 expression**

| Parameter | Patients, *n* (%) | | | *p* value ^a^ |
| --- | --- | --- | --- | --- |
|  | Overall cohort | PTEN_low_-TP53_low_ | Other |  |
| Patients | 42 | 4 (12.1) | 37 (87.9) |  |
| Meets AVPC criteria   Yes   No | 13 (30.9)  29 (69.1) | 2 (50)  2 (50) | 10 (27)  27 (73) | 0.068 |
| Visceral metastasis only | 3 (7.1) | 0 (0) | 3 (8.1) | 1 |
| Lytic bone metastasis | 5 (11.9) | 1 (25) | 4 (10.8) | 0.35 |
| Bulky nodes or prostate mass | 7 (16.7) | 2 (50) | 5 (13.5) | 0.081 |
| Low PSA relative to volume | 4 (9.5) | 1 (25) | 3 (8.1) | 0.13 |
| NE markers + elevated LDH or CEA | 5 (11.9) | 1 (25) | 4 (10.8) | 0.35 |
| Primary castration resistance | 3 (7.1) | 1 (25) | 2 (5.4) | 0.27 |
| Small-cell carcinoma morphology | 4 (9.5) | 1 (25) | 3 (8.1) | 0.29 |

AVPC = aggressive-variant prostate cancer; CEA = carcinoembryonic antigen; LDH = lactate dehydrogenase; NE = neuroendocrine; PSA = prostate-specific antigen.

^a^ *p* values are for Fisher’s exact test.

**Supplementary figures**

**Supplementary Fig. 1 –** Kaplan–Meier CRPC-FS and OS curves according to low expression of combinations of two TSGs: (A) *RB1* and *PTEN*, (B) *RB1* and *TP53*, and (C) *PTEN* and *TP53*.

CRPC-FS = castration-resistant prostate cancer–free survival; m = median months; NR = not reached; OS = overall survival; TSG = tumor suppressor gene. Significant *p* values (<0.05) are indicated in bold font.

**Supplementary Fig. 2 –** Forest plots of multivariate analysis results for CRPC-FS and OS according to low expression of combinations of two TSGs: (A) *RB1* and *PTEN*, (B) *RB1* and *TP53*, and (C) *PTEN* and *TP53*.

CI = confidence interval; CRPC-FS = castration-resistant prostate cancer–free survival; LDH = lactate dehydrogenase; OS = overall survival; TSG = tumor suppressor gene.

**Supplementary Fig. 3 –** (A) TURP sample from a patient with CRPC. The tumor is composed of sheets, trabeculae, nests, and an acinar growth pattern of small or intermediate-sized cells with scant cytoplasm and hyperchromic nuclei. Neoplastic cells show nuclear molding, inconspicuous nucleoli, and evenly dispersed finely stippled chromatin. Apoptotic cells and apoptotic bodies are also evident. Tumor cells show positive immunostaining for synaptophysin. (B) Liver biopsy from another patient with CRPC showing sinusoidal infiltration by neoplastic cells with cytologic features of small cell carcinoma. The presence of clusters and small isolated cells with nuclear molding, scant cytoplasm, and nuclear hyperchromasia is evident. Tumor cells show positive immunostaining for synaptophysin.

CRPC = castration-resistant prostate cancer; H&E = hematoxylin-eosin; IHC = immunohistochemistry; SYP = synaptophysin (antibody: CONFIRM anti-sypnatophysin, SP11; Roche); TURP = transurethral resection of the prostate.
